# Preseason neuromuscular versus endurance training leads to greater improvements in isokinetic strength, muscle damage, and inflammation parameters in highly trained female soccer players

**DOI:** 10.1186/s13102-025-01154-x

**Published:** 2025-04-30

**Authors:** Ali Belamjahad, Claire Tourny, Anthony C. Hackney, Nidhal Jebabli, Naoual Chariba, Fatiha Laziri, Ayoub Saeidi, Ismail Laher, Urs Granacher, Hassane Zouhal

**Affiliations:** 1 Department of Sport Sciences, University of Rouen, Rouen, France; 2 Department of Exercise & Sport Science, Department of Nutrition, University of North Carolina, Chapel Hill, NC USA; 3 Department of Biology and Medical Analysis, Derrak Hospital, Berkane, Morocco; 4 High Institute of Sport and Physical Education of Kef, UR22JS01, University of Jendouba, Kef, Tunisia; 5 Laboratoire Ecologie, Environnement et Santé Equipe Santé Humaine et Environnement, Faculté des Sciences de, Université Moulay Ismail, Meknès, Morocco; 6 Department of Physical Education and Sport Sciences, Faculty of Humanities and Social Sciences, University of Kurdistan, Sanandaj, Kurdistan Iran; 7 Department of Anesthesiology, Pharmacology and Therapeutics, The University of British Columbia, Vancouver, Canada; 8 Department of Sport and Sport Science, Exercise and Human Movement Science, University of Freiburg, Freiburg, Germany; 9 Université Rennes, M2S (Laboratoire Mouvement, Sport, Rennes, Santé France; 10 Institut International des Sciences du Sport (2I2S), Irodouer, 35850 France

**Keywords:** Football, Season, Training, Torque, Power

## Abstract

**Background:**

The preseason offers an opportunity to achieve an optimal level of physical fitness for the entire season. The question arises whether the training programs induce muscle damage and inflammatory processes which may have a negative impact on players’ readiness at the beginning of the season.

**Objectives:**

To examine the effects of a preseason neuromuscular training program (NMT) versus endurance training (ET) on measures of isokinetic strength, muscle damage, and blood parameters in female soccer players.

**Methods:**

Twenty-two highly trained female soccer players with a mean age of 17.0 ± 1.3 years were randomly assigned to a NMT (*n* = 11) or ET group (*n* = 11). NMT and ET programs lasted six weeks with three sessions per week, each 45–60 min. NMT included strength, power, linear sprint and change-of-direction speed, and dynamic stability exercises. ET comprised running, circuit drills, coordination, aerobic circuit, interval-training, speed-endurance exercises, and dynamic stretching. isokinetic knee flexor/extensor parameters (e.g., peak torque) were tested pre- and post-training. Blood samples were analyzed for muscle damage markers: creatine kinase (CK), lactate dehydrogenase (LDH), and inflammation markers: C-reactive protein (CRP), and interleukin-6 (IL-6).

**Results:**

Significant group-by-time interactions were found for all isokinetic parameters (0.001 < *p* < 0.012, 1.35 < d < 4.17). Post-hoc tests revealed significant improvements following NMT but not ET (0.001 < *p* < 0.045, 0.81 < d < 2.46). Additionally, there were also significant group-by-time interactions for IL-6 (*p* = 0.005; d = 0.31), CK (*p* = 0.026; d = 1.0), and LDH (*p* < 0.003; d = 1.44). Variations in IL-6, CK, and LDH determined by post-hoc tests indicated decreases in NMT but not ET (IL-6: *p* = 0.005, d = 1.27; CK: *p* = 0.023, d = 1.01; LDH: *p* = 0.002, d = 1.42).

**Conclusions:**

Six weeks of preseason NMT produced larger improvements in isokinetic strength and less muscle damage and inflammation in highly-trained female soccer players compared to ET group.

**Supplementary Information:**

The online version contains supplementary material available at 10.1186/s13102-025-01154-x.

## Introduction

Soccer is the most practiced and popular sport in the world [1]. It is widely recognized as a high-intensity intermittent activity that requires players to undertake metabolically demanding efforts over the duration of a 2 × 45 min match [2, 3]. Besides aerobic and anaerobic demands, explosive activities such as jumps, sprints, and rapid changes-of-direction (CoD) are required for successful soccer performance [3].

Over the past years, the physical demands of female soccer players have increased enormously with approximately 9 to 11 km of total distance per match, 30% of which is high intensity with over 1,300 changes in activity patterns, 423 accelerations and 430 decelerations [4]. Female soccer players’ level of physical fitness is related to match performance [5]. Accordingly, training related physical fitness development becomes more important in female soccer. The preseason can be used to lay a foundation of physical fitness to be used during the entire season [3, 4]. Traditionally, soccer coaches implement preseason training programs such as endurance training (ET) to lay a solid foundation of aerobic capacity for the entire soccer season. Previously, ET programs applied in soccer focused on low-intensity continuous exercises such as steady-state or variable-paced running, combined with moderate- and high-intensity interval training bouts [6]. There is ample evidence in the literature indicating that particularly high-intensity interval training has the potential to improve players’ physical performance [6].

These training-related challenges highlight the importance of training and testing measures of physical fitness such as muscle strength, linear sprint and CoD speed before and after the preseason. In this context, it is not only important to test and train fitness from a performance perspective but also from an injury preventive point of view. In fact, there is evidence that the knee flexors (hamstrings) are most commonly affected by muscle injuries in soccer [7]. This muscle group plays an essential role in the stability of the knee joint through balanced agonist-antagonistic activation of the thigh muscles. For instance, when shooting a ball, the quadriceps produces muscle strength which is balanced by the eccentric muscle strength of the hamstrings [8]. Indeed, the hamstring muscle group makes it possible to decelerate the extension of the knee and control the excessive contractions of the quadriceps. These active muscle actions protect the ligaments by avoiding anterior tibial translation to ensure knee stability [8, 9].

Accordingly, additionally applied neuromuscular training programs during the preseason may have positive effects on performance development and injury prevention. At the same time, the additional and often high-intensity training contents may induce muscle damage or inflammatory processes due to overreaching or overtraining which could hinder players’ readiness at the start of the season. Of note, training-induced adaptations are mainly explained by physiological stressors causing disorders of the homeostasis [10] and thus muscle damage and inflammatory responses [11]. Previous studies have shown, that intensive soccer training applied during the preseason increases markers of inflammation and muscle damage [12, 13]. The increase in creatine kinase (CK) and lactate dehydrogenase (LDH) activity at the end of the preseason has often been linked with damage of the muscle cells (myocyte) following a period of intense training, incomplete recovery and increased oxidative stress [14]. This increase in muscle damage markers can be explained by the changes at the level of the muscle cell membrane due to the effect of hypoxia and muscle ischaemia caused by the intensive soccer training period, and by the increase in intracellular calcium which would activate calcium-dependent proteases [15].

Neuromuscular training (NMT) is a multimodal intervention program including sprint, CoD, balance, muscle strength and power exercises. NMT has proven to be highly effective in enhancing physical fitness components such as muscle strength and power as well as linear sprint and CoD speed in male and female soccer players [16]. In a previous study, the same group of researchers has shown that the integration of 45 to 60 min of preseason NMT, three times per week, for 6 weeks, can improve physical fitness and prevent injuries in highly trained female soccer players [6].

Accordingly, this study, aimed to examine the impact of a preseason NMT program versus an endurance training (ET) program on isokinetic muscle strength and blood markers in highly trained female soccer players. Based upon the relevant literature [17–21], we hypothesized that particularly NMT improves isokinetic strength parameters more than ET and leads to less muscle damage and inflammation in highly trained female soccer players. This article is a companion to an earlier published article [6] involving the same participant sample, as well as incorporating similar methods and procedures of the aforementioned article. However, the key research outcomes are different between the earlier work and the present study.

## Methods

### Participants

**Fig. 1:**
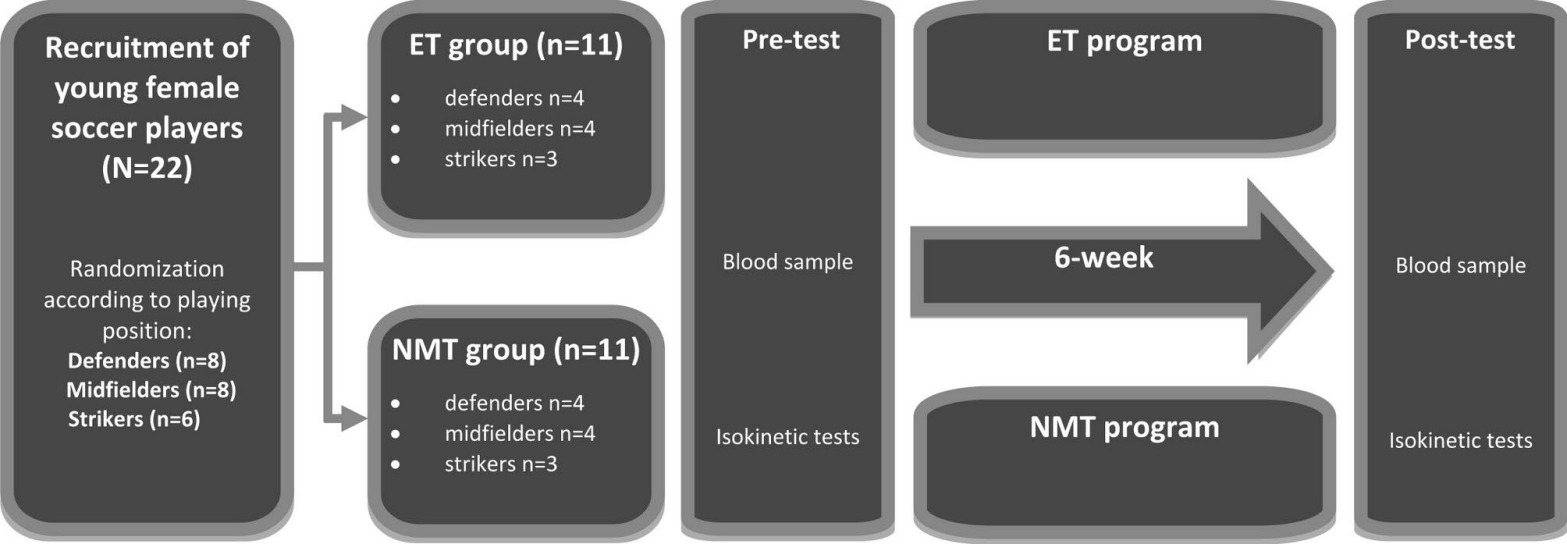
Study design ET, endurance-dominated training; NMT, neuromuscular training; n, number of participants

Twenty-two U19 female soccer players were recruited from a professional soccer club (Renaissance sportive de Berkane 1st Division) to participate in this study. According to the classification of McKay et al., [22], our athletes are ranked at a highly trained level. The anthropometric data of the participants are presented in Table 1. Participants were randomly assigned by their playing position into one of the two intervention groups (NMT, ET) (Fig. 1). Overall, this study included eight defenders, eight midfielders, and six strikers. Goalkeepers were excluded because they already received a position-specific preseason conditioning program. Irrespective of group allocation, all participating players additionally performed five weekly technical and tactical soccer training sessions. The participating players typically trained between ten and eleven months per year, with overall six to eight weekly training sessions and one match per week on the weekend. During the four week off-season period, players performed a strength and conditioning program with three weekly sessions, primarily consisting of general muscle strengthening exercises and aerobic training at low volume and intensity. All players and their parents were advised of the study protocol, potential risks as well as benefits of the study before signing the informed consent. The study utilized and incorporated the ethical standards of the Declaration of Helsinki and was approved by the Local Ethics Committee for Biomedical Research of Oujda (CERBO; ethical approval code: 05/2022), Morocco.

**Table 1:** Characteristics of the study participants

Group	Number	Age (years)	Body height (cm)	Body mass (kg)	Body fat (%)	BMI (kg/m²)
ET	*n* = 11	17.0 ± 1.5	166.3 ± 5.4	60.6 ± 5.7	22.6 ± 3.1	21.9 ± 2.0
NMT	*n* = 11	17.0 ± 1.2	164.9 ± 5.1	57.7 ± 4.5	20.7 ± 3.4	21.2 ± 1.3

ET, endurance training group; NMT, neuromuscular training group; Data are presented as mean ± standard deviation; BMI, body mass index

An a priori power analysis was computed to determine the required sample size (G∗Power, Version 3.1, University of Dusseldorf, Germany) (see earlier published companion article [6]). The analysis indicated that a total sample size of *N* = 20 players would be required to achieve significant group-by-time interactions, given the above-specified conditions. The sample size was increased by 10% (from 20 to 22) to allow for potential dropouts of study participants.

### Estimation of the menstrual cycle phases

We estimated the menstrual cycle phase of each player using the calendar calculation method [23]. The players had to specify the start of their menstruation for the three menstrual cycles preceding the study. From this, we calculated the average cycle length of each player. We assumed the players to have a regular ovulatory menstrual cycle if the standard deviation of the length of each cycle did not exceed three days [23]. During the experiment and the pre/post-test the phases of the menstrual cycle were noted (Table 2) and divided according to the methodological recommendations of Elliott-Sale et al. [24] into the early follicular phase (day 1 to 4), the late follicular phase (day 10 to 13), and the mid-luteal phase (day 20 to 23) based on an average cycle of 28 days [25]. We did not, however, confirm that our participants were ovulatory during the study.

**Table 2:** The phases of the menstrual cycle during pre and post tests

	Group	Pre-test					Post-test			
		EF phase	LF phase	ML phase	Other phases		EF phase	LF phase	ML phase	Other phases
**Isokinetic testing**	ET	2 (18%)	2 (18%)	5 (46%)	2 (18%)		0 (0%)	2 (18%)	4 (36%)	5 (46%)
	NMT	3 (27%)	1 (9%)	6 (55%)	1 (9%)		1 (9%)	2 (18%)	5 (46%)	3 (27%)
**Total**		**5 (22%)**	**3 (14%)**	**11 (50%)**	**3 (14%)**		**1 (5%)**	**4 (18%)**	**9 (41%)**	**8 (36%)**

ET, endurance-dominated training group; NMT, neuromuscular training group; EF, Early follicular; LF, Late follicular; ML, Mid-luteal

### Isokinetic testing of the knee flexors and extensors

Laboratory-based tests were applied before (pre) and after (post) the six-week intervention to determine the effects of NMT versus ET on players’ physical fitness and physiological responses. Isokinetic tests were scheduled during the week preceding the study. A 15-minute warm-up consisting of running and dynamic stretching was scheduled before the tests started. The isokinetic tests were performed and monitored by the same examiner. The tests were performed in the same test sequence and at the same time of day pre- and post- training.

Peak torque of the knee extensors and flexors was obtained on an isokinetic dynamometer (Con-Trex^®^ MJ isokinetic dynamometer, Schnaittach, Germany). We followed the isokinetic strength testing protocol as described by Cotte and Ferret [26]. The female soccer players were placed in an upright seated position on the adjustable Con-Trex^®^ MJ isokinetic dynamometer chair with the hip joint at 100°, the motor axis aligned to the knee joint axis and the arms placed in neutral position alongside the body. They were firmly stabilized through belts around the torso, pelvis, and thigh (1/3 distal) to limit the compensatory movements so that only the knee to be tested was movable with a single degree of freedom. The machine’s lever arm was attached to the lower leg and standardized at 0.30 m in relation to the axis of rotation of the dynamometer [26]. The players performed a warm-up on a bike ergometer for 5 min at an intensity of 75 to 100 W before the test started [27]. Thereafter, the concentric peak torque of the extensor muscles (quadriceps) and the flexor muscles of the knee (hamstrings) were evaluated in three maximal trials at 60 °/s, with five maximum repetitions at 240 °/s [28].

The eccentric torque of the flexors was evaluated in three maximal trials at 30 °/s. Three trials were conducted at each angular velocity. The test results were expressed in absolute (Nm) and relative (Nm/kg) values, with the best peak torque value of the three repetitions defined as maximal strength. A recovery of one minute between sets was allowed. This procedure was performed for both the dominant leg (DL) and the non-dominant leg (NDL). The players were asked to perform a maximal contraction during the test. A conventional and mixed ratio was calculated to identify unilateral lower limb strength imbalances. The conventional hamstring concentric / quadriceps concentric (Hcon / Qcon) ratio was estimated at 240 °/s. and the mixed ratio hamstring eccentric (Hecc, 30 °/s) **/** Qcon (240 °/s) was also measured. The procedure described by Croisier et al. [28] was used to determine the pathological threshold for conventional and mixed ratios. The conventional ratio should be > 0.45 and the mixed ratio should be > 0.89 [28]. The ICCs for test–retest trials for all indices of maximal torque (30, 60, 240 °/s) was between 0.872 and 0.868.

Additionally, the “time to peak torque” of the knee flexors and extensors was analyzed for the three angular speeds (30, 60, 240 °/s), and was defined as the time taken for the muscle to reach its peak torque during extension or flexion and is considered a reliable indicator of the explosive quality of players and is used to evaluate muscle performance [29]. It is important to note that a longer time to peak torque can be a risk factor for Anterior cruciate ligament (ACL) injuries in female soccer players [30]. Time to peak torque is often estimated using an isokinetic dynamometer or force platform to assess muscular performance of a specific movement [29].

### Blood samples

Blood samples were taken pre- and post-training to analyze markers of muscle damage (CK and LDH) and inflammation (CRP and IL-6). The first blood sample was taken prior to the start of the study and the second sample at the end of the six-week training program. Venous blood samples (15 ml) were collected within a 24-hour period before and after the training protocol. The samples were taken between 9:00 a.m. and 10:30 a.m., after an overnight fast and after having not exercised for 12 h from an antebrachial arm vein, using a standard venipuncture technique collected with an ethylenediaminetetraacetic acid (EDTA) tube. Samples were centrifuged for 10 min at 4 °C and 3000 rpm to obtain plasma, and CRP, CK, and LDH activities were determined using a multiparametric analyzer (Konelab 30TM, Thermo Electron Corporation, Holliston, USA). CRP activity was determined using an immunoturbidimetry method, with an intra-assay coefficient of variation of 1.7% for the CRP kit. CK activity was determined with a UV method (IFCC) using N-acetyl-cysteine, and the intra-assay coefficient of variation for the CK kit was 1.8%. LDH activity was determined by applying the enzymatic rate method (IFCC), with an intra-assay coefficient of variation of 1.1% for the LDH. IL-6 activity was determined using the immune-chemiluminescence method (Roche clia cobas e411).

### Training programs

Details on the contents of the training programs can be found in our previously published article [6]. In brief, players completed between six to eight weekly training sessions over the course of the six-week preseason training period. The training sessions were scheduled in the morning at approximately 9 AM (lasting ~ 45–60 min) and in the afternoon at approximately 5 PM (lasting ~ 60–80 min). A minimum of 48 h of rest was granted between the training sessions for both groups. *Training volumes were similar between the two intervention groups (ET vs. NMT) which was realized through similar session duration.* Over the course of the six week training program, training volumes and intensities were gradually increased to avoid overtraining or increase the risk of sustaining injuries. All training sessions were supervised by a professional strength and conditioning coach. The strength and conditioning coach monitored participants to ensure they performed the exercises with correct movement techniques (e.g., lower limb alignment).

### Endurance-dominated training (ET)

The participants assigned to the ET group (*n* = 11) followed a traditional six weeks ET program with three weekly sessions, each session lasted 45–60 min. The training emphasized aerobic capacity during weeks 1, 2, and 3. Thereafter, speed endurance was developed during weeks 4, 5, and 6. More detailed information on the training program can be found in the companion article [6].

### Neuromuscular training (NMT)

The participants assigned to the NMT group (*n* = 11) exercised for six weeks with three weekly sessions (Table 3), each lasting 45–60 min. NMT included a warm-up (joint mobility, high knees, butt kicks, hip rotation, side skips, backward run and shuttle runs), muscle strengthening exercises (half-squat, forward lunge, nordic hamstring, copenhagen exercise, calve extension and hip thrust), plyometrics (forward leap over a 25 and 45 cm obstacle, side jump over cone obstacle and side jump over 35 cm obstacle), CoD speed (180° shuttle runs, 45° and 90° COD speed), and dynamic stability exercises (proprio BOSU ball, plank on swissball, side plank with legs apart, crunch on swissball and lumbar extension). More detailed information on NMT programming can be found in the companion article [6].

**Table 3:** Neuromuscular training program

	Distance	Repetition/Time					
**Warm-up (7’ and 10’)**							
- Joint mobility (Spine, hip, knee, ankle)	2 × 15 s per joint						
- High knees, reps	10 m	2					
- Butt kicks, reps	10 m	2					
- Hip rotation, reps	10 m	2					
- Side skips, reps	10 m	2					
- Backward run, reps	10 m	2					
- Shuttle runs, reps	5–5 m	2					
**Strength (10’ and 15’)**							
- Half-squat, reps	2 × 15		3 × 15	4 × 15	3 × 15	3 × 15	1 × 15
- Forward lunge, reps	10 m	2	3	4	3	3	1
- Nordic hamstring, reps	4		6	8	6	6	4
- Copenhagen exercise (right side), s	2 × 15 s		2 × 20 s	2 × 30 s	2 × 30 s	2 × 20 s	1 × 15 s
- Copenhagen exercise (left side), s	2 × 15 s		2 × 20 s	2 × 30 s	2 × 30 s	2 × 20 s	1 × 15 s
- Calve extension, reps	2 × 15		3 × 15	4 × 15	4 × 15	3 × 15	1 × 15
- Hip thrust, reps	2 × 15		3 × 15	4 × 15	4 × 15	3 × 15	1 × 15
**Plyometrics (7’ to 10’)**							
**-** Forward leap over a 25 cm obstacle, reps			1 × 6	2 × 6	3 × 6	2 × 6	2 × 6
- Side jump over a cone obstacle (right), reps			1 × 6	2 × 6	3 × 6	2 × 6	2 × 6
- Side jump over a cone obstacle (left), reps			1 × 6	2 × 6	3 × 6	2 × 6	2 × 6
- Forward leap over a 45 cm obstacle, reps			1 × 4	2 × 4	3 × 4	2 × 4	2 × 4
- Side jump over 35 cm obstacle (right), reps			1 × 4	2 × 4	3 × 4	2 × 4	2 × 4
- Side jump over 35 cm obstacle (left), reps			1 × 4	2 × 4	3 × 4	2 × 4	2 × 4
**Change-of-direction speed (7’ to 10’)**							
- shuttle runs (angle 180°)	10 m	1	2	3	4	2	1
- change-of-direction speed (angle 45°)	20 m	1	2	3	4	2	1
- change-of-direction speed (angle 90°)	20 m	1	2	3	4	2	1
**Core stability and balance (7’ to 15’)**							
- proprio bosu ball (right leg), s		2 × 15 s–15 s	3 × 20 s–10 s	3 × 30 s–10 s	2 × 45 s–15 s	2 × 45 s–15 s	1 × 45 s–15 s
- proprio bosu ball (left leg), s		2 × 15 s–15 s	3 × 20 s–10 s	3 × 30 s–10 s	2 × 45 s–15 s	2 × 45 s–15 s	1 × 45 s–15 s
- plank on swissball, s		2 × 15 s–15 s	3 × 20 s–10 s	3 × 30 s–10 s	2 × 45 s–15 s	2 × 45 s–15 s	1 × 45 s–15 s
- side plank with legs apart (Right side), s		2 × 15 s–15 s	3 × 20 s–10 s	3 × 30 s–10 s	2 × 45 s–15 s	2 × 45 s–15 s	1 × 45 s–15 s
- side plank with legs apart (Left side), s		2 × 15 s–15 s	3 × 20 s–10 s	3 × 30 s–10 s	2 × 45 s–15 s	2 × 45 s–15 s	1 × 45 s–15 s
- crunch on swissball, s		2 × 15 s–15 s	3 × 20 s–10 s	3 × 30 s–10 s	2 × 45 s–15 s	2 × 45 s–15 s	1 × 45 s–15 s
- lumbar extension, s		2 × 15 s–15 s	3 × 20 s–10 s	3 × 30 s–10 s	2 × 45 s–15 s	2 × 45 s–15 s	1 × 45 s–15 s

Reps, repetitions; s, seconds; m, meter;

### Statistical analyses

To confirm the normality of data distributions, the Shapiro–Wilk test was used. All data are presented as means and standard deviations (SDs). A 2 × 2 analysis of variance (ANOVA) was utilized to assess the data, with the factors being, group (NMT, ET) and time (pre, post). If significant group-by-time interaction effects were detected, Bonferroni adjusted post-hoc tests (t-tests) were computed. Cohen’s d was calculated to quantify meaningful differences for effect size in the data. The interpretation of effect size were; trivial (d < 0.2), small (0.2 < d < 0.5), medium (0.5 < d < 0.8), large (d > 0.8) [31]. Test re-test reliability of the variables was assessed using Cronbach’s model of ICCs and SEMs according to the method previously described [32]. Statistical significance was set at a p level of < 0.05. Data were analyzed using SPSS software (SPSS, version 22, Chicago; IL).

## Results

At pre-test, no significant between-group differences were found for any of the parameters measured. All participants completed their training as prescribed. No training or test-related injuries occurred over the course of the study.

### Isokinetic testing

Results of the isokinetic strength assessment are summarized in Table 4.

**Table 4:** Effects of 6 weeks of neuromuscular (NMT) vs. endurance-dominated (ET) training on isokinetic peak torque (means ± SDs)

Variables	Group	Pre	Post	Change %	Cohen’s d	ANOVA *p*-value (Cohen’s d)		
						Time	Group	Interaction
**Peak torque**								
**Extensors 60°/s conc. DL**								
Peak torque (Nm) (Nm/kg)	ET	117.4 ± 14.7 1.9 ± 0.3	132.5 ± 13.4 2.2 ± 0.2	13.83	1.14	**< 0.001(6.78)**	**0.014(1.24)**	**0.008(2.24)**
(Nm) (Nm/kg)	NMT	119.3 ± 16.7 2 ± 0.3	156.7 ± 16 2.7 ± 0.3	31.31	2.28			
**Extensors 60°/s conc. NDL**								
Peak torque (Nm) (Nm/kg)	ET	107.7 ± 15.8 1.8 ± 0.2	129.2 ± 16.1 2.2 ± 0.2	19.96	1.34	**< 0.001(5.06)**	**0.049(1.47)**	**0.012(2.12)**
(Nm) (Nm/kg)	NMT	108.8 ± 16.1 1.9 ± 0.3	150.4 ± 15.3 2.6 ± 0.3	38.18	2.64			
**Flexors 60°/s conc. DL**								
Peak torque (Nm) (Nm/kg)	ET	75.6 ± 7.8 1.2 ± 0.1	83.3 ± 10.7 1.4 ± 0.1	10.25	0.83	**< 0.001(4.66)**	**0.02(2.39)**	**< 0.001(3.23)**
(Nm) (Nm/kg)	NMT	74.8 ± 9.1 1.3 ± 0.1	100.7 ± 7.1 1.7 ± 0.1	34.65	3.16			
**Flexors 60°/s conc. NDL**								
Peak torque (Nm) (Nm/kg)	ET	72.7 ± 6.4 1.2 ± 0.1	80 ± 8.1 1.3 ± 0.1	9.96	0.99	**< 0.001(1.17)**	**0.046(0.89)**	**< 0.001(2.43)**
(Nm) (Nm/kg)	NMT	70.3 ± 7 1.2 ± 0.1	93.5 ± 7.7 1.6 ± 0.1	32.97	3.13			
**Extensors 240°/s conc. DL**								
Peak torque (Nm) (Nm/kg)	ET	84.1 ± 9.3 1.4 ± 0.2	91.4 ± 11.5 1.5 ± 0.2	8.74	0.70	**< 0.001(3.86)**	**0.037(1.04)**	**< 0.001(2.82)**
(Nm) (Nm/kg)	NMT	82.2 ± 7 1.4 ± 0.1	106.8 ± 5.2 1.8 ± 0.1	29.84	3.96			
**Extensors 240°/s conc. NDL**								
Peak torque (Nm) (Nm/kg)	ET	79.5 ± 9.4 1.3 ± 0.2	89 ± 11.1 1.5 ± 0.2	11.95	0.92	**< 0.001(2.51)**	**0.041(1.13)**	**< 0.001(2.89)**
(Nm) (Nm/kg)	NMT	80. 9 ± 9.6 1.40 ± 0.14	102.6 ± 5.9 1.78 ± 0.12	26.92	2.71			
**Flexors 240°/s conc. DL**								
Peak torque (Nm) (Nm/kg)	ET	53 ± 9.5 0.88 ± 0.14	59.7 ± 9.9 1.01 ± 0.17	12.70	0.69	**< 0.001(2.67)**	**0.004(1.03)**	**< 0.001(3.11)**
(Nm) (Nm/kg)	NMT	55.1 ± 12 0.95 ± 0.21	82.7 ± 8.5 1.43 ± 0.17	50.24	2.65			
**Flexors 240°/s conc. NDL**								
Peak torque (Nm) (Nm/kg)	ET	51.4 ± 9.1 0.85 ± 0.12	59.6 ± 13.9 1.00 ± 0.21	15.95	0.69	**< 0.001(3.11)**	**0.023(1.06)**	**< 0.001(2.28)**
(Nm) (Nm/kg)	NMT	53.4 ± 9.4 0.92 ± 0.15	78.2 ± 11.2 1.36 ± 0.23	46.45	2.40			
**Extensors 30°/s ecc. DL**								
Peak torque (Nm) (Nm/kg)	ET	105.9 ± 14.1 1.78 ± 0.31	117.6 ± 13.9 1.99 ± 0.33	11.05	0.83	**< 0.001(10.20)**	**0.031(0.70)**	**0.007(1.49)**
(Nm) (Nm/kg)	NMT	104.7 ± 11.1 1.81 ± 0.21	134.1 ± 5.5 2.35 ± 0.15	28.01	3.34			
**Extensors 30°/s ecc. NDL**								
Peak torque (Nm) (Nm/kg)	ET	100.1 ± 15.6 1.67 ± 0.27	107 ± 13.5 1.81 ± 0.31	6.83	0.47	**< 0.001(3.61)**	**0.028(0.64)**	**0.003(1.35)**
(Nm) (Nm/kg)	NMT	99.8 ± 12.1 1.73 ± 0.23	128.7 ± 13.5 2.23 ± 0.25	28.92	2.25			
**Flexors 30°/s ecc. DL**								
Peak torque (Nm) (Nm/kg)	ET	150.9 ± 33.9 2.54 ± 0.68	163.2 ± 42.3 2.77 ± 0.83	8.15	0.32	**< 0.001(2.92)**	**0.016(1.24)**	**< 0.001(4.17)**
(Nm) (Nm/kg)	NMT	147.8 ± 28.2 2.54 ± 0.42	233.4 ± 33 4.03 ± 0.55	57.82	2.78			
**Flexors 30°/s ecc. NDL**								
Peak torque (Nm) (Nm/kg)	ET	145.3 ± 35.3 2.45 ± 0.70	162.4 ± 42.6 2.76 ± 0.83	11.76	0.44	**< 0.001(3.97)**	**0.022(0.78)**	**< 0.001(1.46)**
(Nm) (Nm/kg)	NMT	146 ± 28.7 2.52 ± 0.44	222.4 ± 18 3.85 ± 0.30	52.30	3.18			
**Time to peak torque**								
Extensors 60°/s conc. DL	ET	0.61 ± 0.12	0.59 ± 0.16	-3.28	0.14	**0.012(1.81)**	0.364(0.94)	**0.044(1.81)**
	NMT	0.64 ± 0.11	0.48 ± 0.06	-25	1.81			
Extensors 60°/s conc. NDL	ET	0.64 ± 0.11	0.56 ± 0.09	-12.50	0.80	**< 0.001(2.26)**	0.673(0.59)	**0.044(1.20)**
	NMT	0.66 ± 0.07	0.51 ± 0.10	-22.73	1.74			
Flexors 60°/s conc. DL	ET	0.42 ± 0.07	0.36 ± 0.07	-14.29	0.86	**< 0.001(3.61)**	0.133(0.32)	0.133(0.15)
	NMT	0.40 ± 0.06	0.30 ± 0.07	-25	1.53			
Flexors 60°/s conc. NDL	ET	0.35 ± 0.07	0.33 ± 0.06	-5.71	0.31	**0.004(1.18)**	0.331(0.42)	**0.038(1.01)**
	NMT	0.36 ± 0.06	0.27 ± 0.07	-25	1.38			
Extensors 240°/s conc. DL	ET	0.27 ± 0.04	0.25 ± 0.03	-7.41	0.57	**< 0.001(3.17)**	0.324(0.91)	**0.044(1.19)**
	NMT	0.28 ± 0.05	0.21 ± 0.03	-25	1.70			
Extensors 240°/s conc. NDL	ET	0.28 ± 0.04	0.24 ± 0.02	-14.29	1.26	**< 0.001(4.41)**	0.954(0.11)	**0.005(1.32)**
	NMT	0.32 ± 0.06	0.20 ± 0.02	-37.50	2.68			
Flexors 240°/s conc. DL	ET	0.21 ± 0.09	0.17 ± 0.02	-19.05	0.61	**0.001(1.96)**	**0.019(1.77)**	0.387(0.89)
	NMT	0.18 ± 0.05	0.11 ± 0.02	-38.89	1.84			
Flexors 240°/s conc. NDL	ET	0.22 ± 0.05	0.18 ± 0.05	-18.18	0.8	**< 0.001(2.33)**	**0.025(1.61)**	0.123(0.54)
	NMT	0.20 ± 0.08	0.11 ± 0.01	-45.00	1.58			
Extensors 30°/s ecc. DL	ET	2.49 ± 0.27	2.33 ± 0.43	-6.43	0.45	**0.022(1.51)**	0.395(1.55)	0.482(1.20)
	NMT	2.46 ± 0.27	2.17 ± 0.36	-11.79	0.91			
Extensors 30°/s ecc. NDL	ET	2.52 ± 0.20	2.44 ± 0.28	-3.17	0.33	**0.005(3.61)**	0.067(0.64)	0.062(1.35)
	NMT	2.49 ± 0.20	2.13 ± 0.37	-14.46	1.21			
Flexors 30°/s ecc. DL	ET	2.00 ± 0.25	1.96 ± 0.33	-2	0.14	**0.027(3.23)**	0.444(0.14)	0.116(0.43)
	NMT	2.03 ± 0.31	1.77 ± 0.14	-12.81	1.08			
Flexors 30°/s ecc. NDL	ET	2.08 ± 0.22	1.96 ± 0.30	-5.77	0.46	**0.021(2.84)**	0.484(0.29)	0.407(0.43)
	NMT	2.08 ± 0.30	1.85 ± 0.16	-11.06	0.96			
**Ratios**								
**Conventional ratio** H conc. 240°/s **/** Q conc. 240°/s								
DL	ET	0.63 ± 0.13	0.65 ± 0.11	3.17	0.17	**< 0.001(2.07)**	0.078(0.49)	**0.013(1.63)**
	NMT	0.66 ± 0.12	0.79 ± 0.08	19.70	1.27			
NDL	ET	0.65 ± 0.13	0.67 ± 0.17	3.08	0.13	**< 0.001(0.90)**	0.3(0.09)	**0.015(1.45)**
	NMT	0.66 ± 0.10	0.77 ± 0.11	16.67	1.05			
**Mixed ratio** H ecc. 30°/s **/** Q conc. 240°/s								
DL	ET	1.80 ± 0.42	1.81 ± 0.53	0.56	0.02	**0.007(2.56)**	0.238(0.82)	**0.008(1.40)**
	NMT	1.79 ± 0.31	2.18 ± 0.29	21.79	1.30			
NDL	ET	1.84 ± 0.46	1.84 ± 0.53	0	0	**< 0.01(2.07)**	0.338(0.79)	**< 0.01(0.82)**
	NMT	1.81 ± 0.34	2.17 ± 0.24	19.89	1.22			

ET, endurance-dominated training group; NMT, neuromuscular training group; conc., concentric; ecc., eccentric; H, hamstring; Q, Quadriceps; DL, Dominant leg; NDL; Non-dominant leg; significant outcomes are highlighted in bold.

Significant group-by-time interactions were observed for peak torque of the knee flexors/ extensors of the DL and the NDL for Conc./Conc. (30°/s; 60°/s; 240°/s; 0.001 < *p* < 0.012, 1.35 < d < 4.17).

Post-hoc analyses showed significant increases in the NMT group for peak torque of DL and NDL knee flexors/extensors in Conc./Conc. mode at 30°/s, 60°/s, and 240°/s angular velocities (*p* ≤ 0.001, 1.34 < d < 2.46). Furthermore, significantly shorter time to reach DL and NDL knee flexor/extensor peak torque in Conc./Conc. mode at 60°/s and 240°/s angular velocities (0.001 < *p* < 0.03, 0.81 < d < 1.11). Additionally, a significant increase was found at post for the conventional ratio H conc. (240°/s) / Q conc. (240°/s) of the DL (*p* = 0.016, d = 1.06), NDL (*p* = 0.049, d = 0.79), and for the mixed ratio H ecc. (30°/s) / Q conc. (240°/s) of the DL (*p* = 0.045, d = 0.86), NDL (*p* = 0.023, d = 1.26).

### Blood responses

The results of the analysis of the markers of inflammation and muscle damage are displayed in Table 5. Significant main time effects were observed for CRP (*p* < 0.001; d = 2.92). There were significant group-by-time interactions for IL-6 (*p* = 0.005; d = 0.31), CK (*p* = 0.026; d = 1.00), and LDH (*p* = 0.003; d = 1.44). Post-hoc tests revealed decreased IL-6, CK, and LDH responses in the NMT but not in the ET group (IL-6: *p* = 0.005, d = 1.27; CK: *p* = 0.023, d = 1.01; LDH: *p* = 0.002, d = 1.42).

**Table 5:** Values for inflammation and muscle-damage markers measured before and after 6 weeks of neuromuscular (NMT) or endurance-dominated (ET) training (means ± SDs)

Variables	Group	Pre	Post	Change %	Cohen’s d	ANOVA *p*-value (Cohen’s d)		
						Time	Group	Interaction
**CRP (mg/L)**	ET	0.84 ± 0.23	2.07 ± 1.07	146.43	0.85	**< 0.001(2.92)**	0.721(0.16)	0.803(0.11)
	NMT	0.87 ± 0.36	2.20 ± 0.79	152.87	0.98			
**IL-6 (pg/mL)**	ET	0.99 ± 0.15	4.43 ± 1.52	347.47	1.83	**< 0.001(0.96)**	**0.008(0.28)**	**0.005(0.31)**
	NMT	1.02 ± 0.11	3.90 ± 0.53	282.35	2.43			
**CK (UI/L)**	ET	95.1 ± 15.7	184.3 ± 32.1	93.9	6.91	**< 0.001(3.66)**	0.06(0.84)	**0.026(1.00)**
	NMT	98.5 ± 20.8	148.3 ± 38.9	50.6	7.50			
**LDH (UI/L)**	ET	99.6 ± 18.0	209.4 ± 25.9	110.3	10.22	**< 0.001(3.77)**	**0.016(1.12)**	**0.003(1.44)**
	NMT	105.4 ± 16.6	168.1 ± 31.9	59.4	9.73			

ET, endurance-dominated training group; NMT, neuromuscular training group; CRP, C-reactive protein; IL-6, interleukin-6; CK, creatine-kinase; LDH, lactate dehydrogenase; significant outcomes are highlighted in bold

### Training load

Specific details on the internal and external training loads were published prior in our companion article [see reference 6].

## Discussion

This study compared the effects of NMT versus ET focused training programs applied during the preseason on isokinetic strength parameters, and blood markers of muscle damage, and inflammation in highly trained female soccer players. The main findings of this study were that the six weeks of specialized NMT showed larger improvements in measures of isokinetic strength, muscle damage, and inflammation blood markers compared with the ET group. It is important to note that the menstrual cycle phases may have an impact on players’ physical performance. However, findings in the scientific literature are controversial with regards to the impact of the menstrual cycle on performance [33]. Previously, authors reported improved linear sprint speed (10, 20, and 30 m) during the late follicular phase and maintenance of lower limb strength and agility during the different phases of the menstrual cycle when performing the vertical jump test (squat-jump) and the 15-m agility test with and without a ball [23]. In the present study, the majority of female soccer players were in the mid-luteal phase during pre and post tests. Furthermore, during the post-test period, only 5% of players, mainly in the NMT group, were in the early follicular phase. Thus, we assume that the menstrual cycle did not have a substantial influence on the physical performance of the players during the testing periods, but we acknowledge we have to be tentative on this point.

### Isokinetic testing

#### Peak torque of the knee extensors and flexors

Findings from this study indicate that knee flexor/extensor peak torque improved at different angular velocities (Con 60°/s, Con 240°/s, Ecc 30°/s) and for DL, NDL ratios in the NMT group only. This improvement can be ascribed to the effects of NMT which strengthens the interplay between the nervous system and the muscles, resulting in improved recruitment and synchronization of motor units. Thus, NMT contributes to a better production of the peak torque [34, 35]. Our results agree with several other investigations which demonstrated that NMT can improve knee extensors and flexors peak torque in soccer players [36–38].

It is important to note that a greater knee extensor and flexor peak torque could allow players to improve their performance of specific movements in soccer, such as sprinting, shooting, and jumping [39]. Morevover, Zhang et al. [35] observed that NMT improved the knee isokinetic peak torques at different angular velocities and allowed for improved balance of peak torque during rapid angular velocity tests. These changes can be related to the specificity of NMT exercises aimed at improving muscle strength and power (explosive actions in the stretch-shortening cycles), jumping, and agility [35].

Furthermore, improving the knee isokinetic peak torque ratio (flexors/extensors) is a vital component of physical fitness and injury prevention. Of note, the NMT program improved the conventional and the mixed ratio [36, 40]. A conventional ratio below 0.6 and a mixed ratio below 1.0 can lead to hamstring and anterior cruciate ligament injuries [28, 29, 36]. Our study reports improvements in the ratios to values higher than the thresholds defined in the literature [28]. A higher ratio, as such, is associated with a longer professional training level and also with high technical quality in soccer [41]. Our findings are in agreement with the results of several other studies that demonstrated that six to eight-week NMT improves the conventional and the mixed hamstring/quadriceps ratios in soccer players [36, 40, 42, 43].

#### Time to peak torque

Our study demonstrates a significant reduction in the time required to reach maximum peak torque in the NMT group compared to the ET group (i.e., at different angular velocities in both limbs, dominant and non-dominant leg). This improvement agrees with other reports that multimodal NMT including plyometrics, dynamic stability, linear sprint and change-of-direction speed exercises improves time to peak torque. In addition, NMT improves time to peak torque by improving strength, power, and muscle activation timing, resulting in faster development of strength to achieve peak torque [44]. Our study reinforces this view that NMT improves aspects of physical fitness related to soccer performance.

#### Blood analysis

Our results show increases in the extent of muscle damage and inflammation from pre to posttest in both study groups, which is in agreement with other findings that a period of preseason or intensive training increased markers of inflammation and muscle damage [45, 46]. These increases can mainly be explained by damage to the muscle cell membrane caused by hypoxia, muscle ischemia after an intensive training period, and also by increases in intracellular calcium levels that can activate calcium-dependent proteases [46].

However, our results contrast with those of Coppalle et al. [47] who reported no differences in biomarkers of inflammation and muscle damage biomarkers in professional male soccer players following two six-week preseason training periods. Specifically, their study found no changes in CK and CRP levels between the start and end of the preseason period (*p* > 0.05), although there were increases in LDH levels during the early season of preparation (*p* = 0.007, d = 0.904) [47]. However, the blood samples in the study by Coppalle et al. [47] were not taken immediately after the end of the preseason, but a week later, which might have been sufficient time for biomarker levels to return to baseline values.

We report that participants in the NMT group had lower levels of muscle damage (CK and LDH) and inflammation (IL-6) compared to the ET group at post tests (i.e., an interaction effect). This could have been due to structural adaptations of the musculoskeletal and nervous systems in response to neuromuscular training [48], as supported by the findings of Clarkson et al. [49] that a single session of eccentric exercise causes muscles to be more resistant to future muscle damage (“preconditioning”). This positive adaptation can improve muscle protection and possibly accounts for the greater reduction of muscle damage in participants in the NMT group compared to those in the ET group.

### Study limitations

We recognize that a limitation of the present study is that we utilized small sample sizes within each research group. Therefore, further research with a larger sample is required to confirm or refute the effects of the NMT versus ET programs we observed. For a better understanding of the underlying physiological mechanisms of NMT-related adaptations in female soccer players, a more rigorous assessment of neuromuscular adaptations using procedures such as electromyography (muscle activation), ultrasound (muscle hypertrophy), or other imaging technology is desirable. Finally, the lack of an objective assessment of the menstrual phase, such as hormonal measurements, must also be considered a weakness within our study.

## Practical applications and conclusions

The results herein indicate that the integration of NMT during the preseason preparation period can be considered an effective approach to improve isokinetic strength parameters. Moreover, NMT compared to ET causes less marked increases in muscle damage and inflammatory responses in highly-trained female soccer players (Tier 3).

## Electronic supplementary material

Below is the link to the electronic supplementary material.

Supplementary Material 1

## Data Availability

The datasets generated during and analyzed during the current study are not publicly available due to confidential information about the participants but are available from the corresponding author on reasonable request.

## Declarations

### Clinical trial number

PACTR202409922230803. https://pactr.samrc.ac.za/Admin/Home.aspx.

### Date of registration

August 7th 2024.

## References

[1] Reilly T, Bangsbo J, Franks A. Anthropometric and physiological predispositions for elite soccer. Sports Sci. 2000;18:669–83. 10.1080/02640410050120050 11043893

[2] Stolen T, Chamari K, Castagna C, Wisloff U. Physiology of soc-cer: an update. Sports Med. 2005;35(6):501–36. 15974635 10.2165/00007256-200535060-00004

[3] Zouhal H, Coppalle S, Rave G, et al. Football de haut-niveau: analyses physique et physiologique–blessures et prévention. Sci Sports. 2021;36:332–57.

[4] Mara JK, Thompson KG, Pumpa KL, Morgan S. The acceleration and deceleration profiles of elite female soccer players during competitive matches. J Sci Med Sport. 2017;20(9):867–72. 28173971 10.1016/j.jsams.2016.12.078

[5] Little T, Williams AG. Specificity of acceleration, maximum speed, and agility in professional soccer players. J Strength Conditioning Res. 2005;19(1):76–8. 10.1519/14253.1 15705049

[6] Belamjahad A, Tourny C, Jebabli N, et al. Effects of a preseason neuromuscular training program vs. an Endurance-Dominated program on physical fitness and injury prevention in female soccer players. Sports Medicine-Open. 2024;10:76. 38922502 10.1186/s40798-024-00731-7 PMC11208342

[7] Ekstrand J, Hägglund M, Waldén M. Epidemiology of muscle injuries in professional football (soccer). Am J Sports Med. 2011;39(6):1226–32. 21335353 10.1177/0363546510395879

[8] Baroni BM, Ruas CV, Ribeiro-Alvares JB, Pinto RS. Hamstring-to-quadriceps torque ratios of professional male soccer players: A systematic review. J Strength Conditioning Res. 2020;34(1):281–93. 10.1519/JSC.0000000000002609. 10.1519/JSC.0000000000002609 29794893

[9] Aicale R, Tarantino D, Maffulli N. Overuse injuries in sport: a comprehensive overview. J Orthop Surg Res. 2018;13(1):1–11. 30518382 10.1186/s13018-018-1017-5 PMC6282309

[10] Coffey VG, Hawley JA. The molecular bases of training adaptation. Sports Med. 2007;37:737–63. 17722947 10.2165/00007256-200737090-00001

[11] Romagnoli M, Sanchis-Gomar F, Alis R, Risso-Ballester J, Bosio A, Graziani RL, Rampinini E. Changes in muscle damage, inflammation, and fatigue-related parameters in young elite soccer players after a match. J Sports Med Phys Fit. 2016;56(10):1198–205. 26558831

[12] Radzimiński Ł, Jastrzębski Z, López-Sánchez GF, Szwarc A, Duda H, Stuła A, Dragos P. Relationships between training loads and selected blood parameters in professional soccer players during a 12-day sports camp. Int J Environ Res Public Health. 2020;17(22):8580. 33227932 10.3390/ijerph17228580 PMC7699258

[13] Malone S, Mendes B, Hugues B, Roe M, Devenney S, Collins K, et al. Decrements in neuromuscular performance and increases in creatine kinase impact training outputs in elite soccer players. J Strength Cond Res. 2018;32:1342–51. 10.1519/JSC.0000000000001997. 28557851 10.1519/JSC.0000000000001997

[14] Becatti M, Mannucci A, Barygina V, Mascherini G, Emmi G, Silvestri E, Fiorillo C. Redox status alterations during the competitive season in élite soccer players: focus on peripheral leukocyte-derived ROS. Intern Emerg Med. 2017;12:777–88. 28361355 10.1007/s11739-017-1653-5

[15] Pimenta E, Coelho DB, Capettini L, Gomes T, Pussieldi G, Ribeiro J, Silami-Garcia E. Analysis of creatine kinase and alpha-actin concentrations in soccer pre-season. Revista Brasileira De Ciência E Movimento. 2015;23(4):5–14.

[16] Radnor JM, Lloyd RS, Oliver JL. (2017). Individual response to different forms of resistance training in school-aged boys. J Strength Cond Res. 2017; 31(3), 787–797. 10.1519/JSC.0000000000001527 27379963

[17] Mujika I, Santisteban J, Impellizzeri FM, Castagna C. Fitness determinants of success in Men’s and women’s football. J Sports Sci. 2009;27:107–14. 19058090 10.1080/02640410802428071

[18] Pardos-Mainer E, Casajús JA, Bishop C, Gonzalo-Skok O. Effects of combined strength and power training on physical performance and interlimb asymmetries in adolescent female soccer players. Int J Sports Physiol Perform. 2020;15:1147–55. 32820132 10.1123/ijspp.2019-0265

[19] Ramírez-Campillo R, Vergara-Pedreros M, Henríquez-Olguín C, et al. Effects of plyometric training on maximal-intensity exercise and endurance in male and female soccer players. J Sports Sci. 2016;34:687–93. 26197721 10.1080/02640414.2015.1068439

[20] Ramirez-Campillo R, García-Pinillos F, García-Ramos A, et al. Effects of different plyometric training frequencies on components of physical fitness in amateur female soccer players. Front Physiol. 2018;9:387092. 10.3389/fphys.2018.00934 PMC6056896 30065665

[21] Roso-Moliner A, Mainer-Pardos E, Cartón-Llorente A, Nobari H, Pettersen SA, Lozano D. Effects of a neuromuscular training program on physical performance and asymmetries in female soccer. Front Physiol. 2023;14:1171636. 37256070 10.3389/fphys.2023.1171636 PMC10226082

[22] McKay AK, Stellingwerff T, Smith ES, Martin DT, Mujika I, Goosey-Tolfrey VL, Burke LM. Defining training and performance caliber: a participant classification framework. Int J Sports Physiol Perform. 2021;17(2):317–31. 10.1123/ijspp.2021-0451 34965513

[23] Igonin P-H, Rogowski I, Boisseau N, Martin C. Impact of the menstrual cycle phases on the movement patterns of sub-elite women soccer players during competitive matches. Int J Environ Res Public Health. 2022;19:4465. 35457332 10.3390/ijerph19084465 PMC9025339

[24] Elliott-Sale KJ, Minahan CL, de Jonge XAJ, et al. Methodological considerations for studies in sport and exercise science with women as participants: a working guide for standards of practice for research on women. Sports Med. 2021;51:843–61. 33725341 10.1007/s40279-021-01435-8 PMC8053180

[25] De Jonge XAJ. Effects of the menstrual cycle on exercise performance. Sports Med. 2003;33:833–51. 12959622 10.2165/00007256-200333110-00004

[26] Cotte T, Ferret J-M. Comparative study of two isokinetics dynamometers: CYBEX NORM vs CON-TREX MJ. Isokinet Exerc Sci. 2003;11:37–43.

[27] Croisier J-L, Forthomme B, Namurois M-H, Vanderthommen M, Crielaard J-M. Hamstring muscle strain recurrence and strength performance disorders. Am J Sports Med. 2002;30:199–203. 11912088 10.1177/03635465020300020901

[28] Croisier J-L, Ganteaume S, Binet J, Genty M, Ferret J-M. Strength imbalances and prevention of hamstring injury in professional soccer players: a prospective study. Am J Sports Med. 2008;36:1469–75. 18448578 10.1177/0363546508316764

[29] Apaydın N, İnce A. Investigation of the hamstrıng/quadriceps ratio, time to peak torque and joint angle at peak torque characteristics of female soccer players. Online J Recreation Sports. 2023;12:435–41.

[30] Fischer DV. Neuromuscular training to prevent anterior cruciate ligament injury in the female athlete. Strength Conditioning J. 2006;28:44–54.

[31] Cohen J. Statistical power analysis for the behavioral sciences. Routledge; 2013.

[32] Cronbach LJ. Coefficient alpha and the internal structure of tests. Psychometrika. 1951;16:297–334.

[33] Carmichael MA, Thomson RL, Moran LJ, Wycherley TP. The impact of menstrual cycle phase on athletes’ performance: a narrative review. Int J Environ Res Public Health. 2021;18(4):1667. 33572406 10.3390/ijerph18041667 PMC7916245

[34] Panagoulis C, Chatzinikolaou A, Avloniti A, et al. In-season integrative neuromuscular strength training improves performance of early-adolescent soccer athletes. J Strength Conditioning Res. 2020;34:516–26. 10.1519/JSC.0000000000002938 30431535

[35] Zhang X, Hu M, Lou Z, Liao B. Effects of strength and neuromuscular training on functional performance in athletes after partial medial meniscectomy. J Exerc Rehabilitation. 2017;13:110. 10.12965/jer.1732864.432 PMC5331991 28349042

[36] Arsenis S, Gioftsidou A, Ispyrlidis I, et al. Effects of the FIFA 11 + injury prevention program on lower limb strength and balance. J Phys Educ Sport. 2020;20:592–8.

[37] Daneshjoo A, Rahnama N, Mokhtar AH, Yusof A. Effectiveness of injury prevention programs on developing quadriceps and hamstrings strength of young male professional soccer players. J Hum Kinetics. 2013;39:115. 10.2478/hukin-2013-0074 PMC3916926 24511347

[38] Impellizzeri FM, Bizzini M, Dvorak J, Pellegrini B, Schena F, Junge A. Physiological and performance responses to the FIFA 11+(part 2): a randomised controlled trial on the training effects. J Sports Sci. 2013;31:1491–502. 23855764 10.1080/02640414.2013.802926

[39] Śliwowski R, Grygorowicz M, Wieczorek A, Jadczak Ł. The relationship between jumping performance, isokinetic strength and dynamic postural control in elite youth soccer players. J Sports Med Phys Fit. 2018;58:1226–33. 10.23736/S0022-4707.17.07289-9 28639440

[40] Brito J, Figueiredo P, Fernandes L, et al. Isokinetic strength effects of FIFA’s the 11+ injury prevention training programme. Isokinet Exerc Sci. 2010;18:211–5.

[41] Śliwowski R, Marynowicz J, Grygorowicz M, Wieczorek A, Jadczak Ł. Are there differences in concentric isokinetic strength perfor-mance profiles between international and non-international elite soccer players? Int J Environ Res Public Health. 2021;18:35. 10.3390/ijerph18010035 PMC7793063 33374580

[42] Dello Iacono A, Padulo J, Ayalon M. Core stability training on lower limb balance strength. J Sports Sci. 2016;34:671–8. 26177151 10.1080/02640414.2015.1068437

[43] Yılmaz AK, Kabadayı M, Bostancı Ö, Yılmaz C, Mayda MH, INFLUENCE OF CORE STRENGTH TRAINING ON PEAK MUSCLE TORQUE OF QUADRICEPS AND HAMSTRING IN YOUNG SOCCER PLAYERS. Kinesiologia Slov. 2020;26.

[44] Besson T, Pastor FS, Varesco G, et al. Elite vs. Experienced male and female trail runners: comparing running economy, biomechanics, strength, and power. J Strength Conditioning Res. 2023;37:1470–8. 10.1519/JSC.0000000000004412 37347946

[45] Radzimiński Ł, Jastrzębski Z, López-Sánchez GF, et al. Relationships between training loads and selected blood parameters in professional soccer players during a 12-day sports camp. Int J Environ Res Public Health. 2020;17:8580. 33227932 10.3390/ijerph17228580 PMC7699258

[46] Pimenta E, Coelho DB, Capettini L, et al. Analysis of creatine kinase and alpha-actin concentrations in soccer pre-season. Revista Brasileira De Ciência E Movimento. 2015;23:5–14.

[47] Coppalle S, Rave G, Ben Abderrahman A, et al. Relationship of pre-season training load with in-season biochemical markers, injuries and performance in professional soccer players. Front Physiol. 2019;10:426414. 10.3389/fphys.2019.00409 PMC6474299 31031638

[48] Podgórski T, Kryściak J, Pluta B, et al. A practical approach to monitoring biomarkers of inflammation and muscle damage in youth soccer players during a 6-Month training cycle. J Hum Kinetics. 2021;80:185–97. 10.2478/hukin-2021-0093 PMC8607760 34868428

[49] Clarkson PM, Nosaka K, Braun B. Muscle function after exercise-induced muscle damage and rapid adaptation. Med Sci Sports Exerc. 1992;24:512–20. 1569847

